# The Outcome of Ventricular Arrhythmias Associated With Mitral Valve Prolapse After Catheter Ablation: A Systematic Review and Meta-Analysis

**DOI:** 10.7759/cureus.20310

**Published:** 2021-12-09

**Authors:** Kevin Wibawa, Ignatius Ivan, Giovanni Jessica, Denio Ridjab

**Affiliations:** 1 Family Medicine, Gunung Jati General Hospital, Cirebon, IDN; 2 Medical Education, Faculty of Medicine and Health Science, Atma Jaya Indonesian Catholic University, Jakarta, IDN; 3 Family Medicine, Saint Elisabeth Hospital, Semarang, IDN

**Keywords:** premature ventricular complex, ventricular fibrillation, ventricular tachycardia (vt), catheter ablation, mitral valve prolapse

## Abstract

The presence of mitral valve prolapse (MVP) varies from asymptomatic to life-threatening arrhythmias. Catheter ablation (CA) is widely used to treat ventricular arrhythmias (VAs) associated with MVP. Despite having high procedural success, outcome data after CA is limited, especially in a long-term setting. Therefore, this systematic review and meta-analysis were performed. Literature searching was conducted in Pubmed, EuropePMC, Proquest, and Ebsco from inception to December 2020 using keywords: ventricular arrhythmia, premature ventricular complex, ventricular tachycardia, ventricular fibrillation, mitral valve prolapse, and catheter ablation. A total of 407 potential articles were retrieved for further review. The final review resulted in six articles for systematic review and meta-analysis. The study was registered in PROSPERO (CRD42020219144). The most common origin of VAs was papillary muscle. The acute success rate of CA in the MVP group varies between 66% and 94%. Follow-up studies reported a higher percentage of VAs recurrence after CA in the MVP group (22.22%) compared with the non-MVP group (11.38%). However, the difference is not significant (P-value = 0.16). Other studies reported a 12.5%-36% rate and 40% of repeat ablation in the medium term and the long term, respectively. Episodes of sudden cardiac death during exertion could still occur following CA in patients with MVP. Distinct origin of VAs was observed during repeated ablation procedures, which may explain arrhythmic substrate progression. Diffuse left ventricular fibrosis around papillary muscle rather than local fibrosis was observed among older patients. Furthermore, the presence of mitral annular disjunction (MAD) and Filamin C mutation might increase the risk of recurrent VAs. CAn has been done as the treatment of VAs associated with MVP. The acute success rate of CA varies between studies and the number of patients requiring repeat CA varied from 12.5% to 40%. Sudden cardiac death could still occur after CA. Older age during CA, genetic predisposition, deep arrhythmic foci, multifocal VAs origin, diffuse fibrosis, and the presence of MAD may contribute to the recurrence of VAs. Further studies, stratification, and evaluation are needed to prevent fatal outcomes in VA associated with MVP, even after CA.

## Introduction and background

The spectrum of ventricular arrhythmias (VAs) varies from asymptomatic premature ventricular complex (PVC) to life-threatening ventricular fibrillation (VF) [1]. Advances in knowledge of VAs provide new insights into the mechanism of the VA. Mitral valve prolapse (MVP) has been highlighted in its relation with VAs. MVP is a condition when one or both of the mitral leaflets abnormally protrude beyond the mitral annulus into the left atrium during ventricular systole [2]. Although MVP is thought of as a benign and stable condition, findings in medical research have shown a new insight and course of the disease [3,4]. The occurrence of non-sustained ventricular tachycardia (NSVT), sustained ventricular tachycardia (VT), PVC, and VF associated with MVP has been reported [5]. The prevalence of MVP is estimated at around 1% to 3% in the general population [6]. The clinical presentation differs between patients with MVP. While one can be an asymptomatic female patient with mid-systolic click and late systolic murmur, the other patient may come with chest pain, palpitation, syncope, or dyspnea [2,7].

MVP is associated with various deleterious complications, such as mitral regurgitation, heart failure, endocarditis, atrial fibrillation, stroke, and VAs [5,7,8]. The VAs are associated with sudden cardiac death (SCD) [5,6]. The prevalence of SCD in young adults with MVP ≤40 years old was 7% from all cases of SCD [5]. One study reported the incidence of PVC among the adult population with MVP is between 49% and 85% [3]. Higher PVC burden was associated with NSVT and sustained VT [9]. In addition, PVC may induce VF in MVP patients. [10,11]. Although the pathophysiology between VAs associated with MVP is not well understood, some hypotheses have been proposed to explain the pathophysiology. The inferolateral base of the left ventricle (LV) or the papillary muscle (PM) is the most vulnerable area to develop fibrosis caused by mechanical traction of protruding leaflets [6]. In addition, the presence of mitral annular disjunction (MAD) can increase wall stress in the inferolateral base of the LV and PM [3]. The mechanical traction of the PM may alter the action potential duration, induce early after depolarization, and abnormal automaticity [6,12]. Consequently, the VAs in patients with MVP needs to be treated.

Catheter ablation (CA) emerges as one of the modalities to treat VA in patients with MVP [6]. Even though the acute procedural success of CA in MVP patients with VAs is quite high, there is a scarcity of data regarding outcomes of CA in VAs associated with MVP, especially in the long term [4,6]. Therefore, this study aims to evaluate the outcomes of VAs associated with MVP after CA.

## Review

Methods

**Figure 1:**
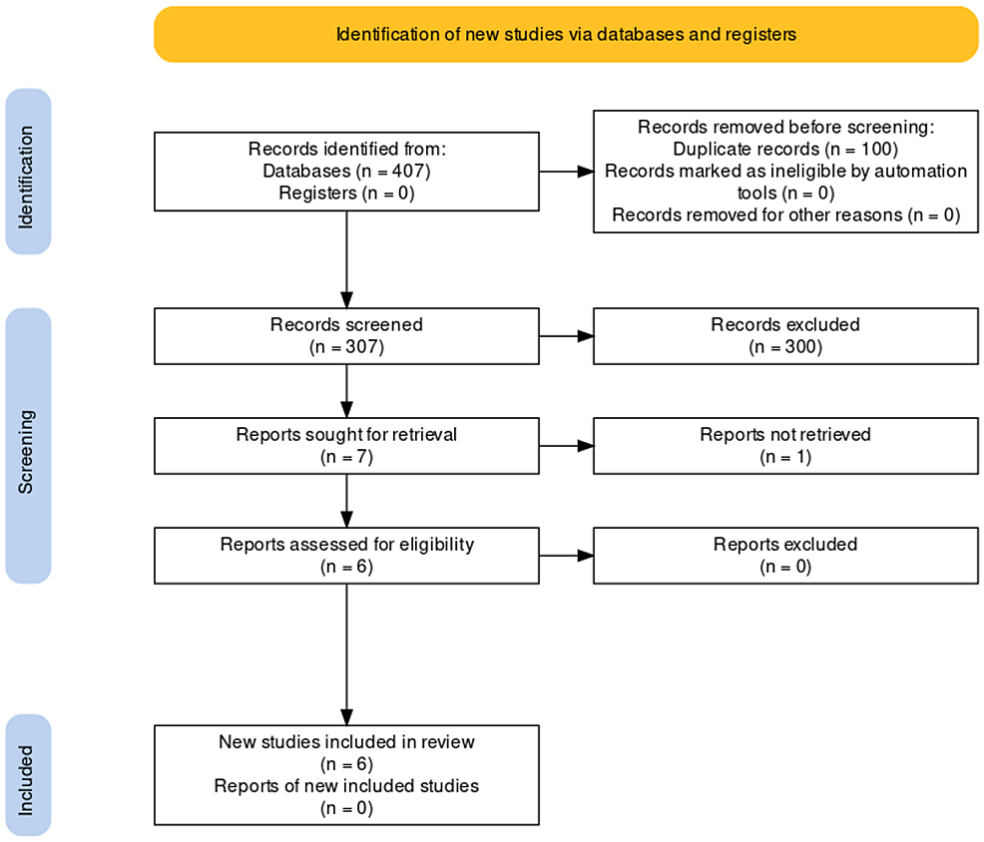
Flowchart of the study selection process

This study is previously registered in the International Prospective Register of Systematic Reviews (PROSPERO) by the following number: CRD42020219144. A structured search of the literature was conducted to identify research on the outcome of VA associated with MVP after CA, using the Preferred Reporting Items for Systematic Reviews and Meta-analyses (PRISMA) statement guideline, with a pre-determined search strategy [13]. The process of study selection can be seen in Figure 1.

The search was conducted in Pubmed, EuropePMC, Proquest, and Ebsco from inception to December 2020. We combined search strategy by using MeSH terms and [Title/Abstract], with the following keywords: ventricular arrhythmia, premature ventricular complex, ventricular tachycardia, ventricular fibrillation, mitral valve prolapse, and catheter ablation. The search was performed by two independent reviewers (KW and II). Any differences were solved through a discussion. Without an agreement, the opinion of the third reviewer (GJ) was consulted to make the final decision.

All results from the search strategy were exported into Endnote X9. Duplicate studies were removed and articles were reviewed based on the title and abstract of the studies. Studies were reviewed and included based on the following criteria: (1) with and without a control group, (2) VAs (PVC, VT, VF), (3) including patients with MVP, and (4) population aged more than 18 years old. The study was excluded if: (1) non-English article, (2) full text failed to be retrieved, (3) study design such as systematic review, meta-analysis, correspondence, case report, case series, literature review, letter to the editor. After included studies were identified, data extraction was performed. The following data will be extracted from the included studies: First author, publication year, study design, country of origin, sample size, baseline participant characteristics, leaflet involvement in MVP, type and origin of VA, type of CA, the acute success rate of MVP group and control group, follow up period and outcome after CA. Authors of studies were contacted via email to request access to missing data. Acute CA success was defined as the absence of the clinical VAs at 30 minutes after the procedure [10]. For summary measurement, we will estimate the prevalence of death, VA, and adverse events in patients with MVP getting CA.

All observational studies were evaluated for their methodological quality using Newcastle Ottawa Scale (NOS). The NOS assessed participant selection, comparability, and outcome reporting using 8 subscale items. In cohort studies, a maximum score of 9 from the sum of subscale items will be used. This critical appraisal were be performed by KW and II. Any disagreements will be resolved through discussion. Without an agreement, the opinion of the third reviewer (GJ) was consulted to make the final decision.

The meta-analysis was performed using Review Manager 5.4. The random-effects model was used assuming there will be considerable heterogeneity. A narrative synthesis will be performed and focuses on the outcome of VAs associated with MVP after CA. Text and tables will be used to provide the summary and findings of the included studies.

Results

A total of 407 articles were retrieved during the initial search. After the exclusion of duplicate articles, 305 potential articles were retrieved for a title and abstract screening. Irrelevant articles, consensus, literature review, systematic review, meta-analysis, case report, case series, commentary, articles with non-human subjects, and non-English articles were excluded. After title and abstract screening, seven articles were included for full-text assessment. One article was excluded because the full text could not be retrieved. The remaining six final articles were included for further analysis in this study [4,10,14-17]. All the articles were retrospective studies. Only three articles compared VAs in the MVP group and the non-MVP group who underwent CA. All articles used radiofrequency CA (RFCA) for the ablation of VAs.

Quality assessment of included retrospective studies is presented in Table 1. Most of the retrospective studies had adequate cohort selection, comparability, and outcome assessment. One study did not have a control group and another study had subjects lost to follow <20%.

**Table 1:** Quality assessment of the included studies

Author	Selection				Comparability	Outcome			Total score
	Representative of the exposed cohort	Selection of the non-exposed cohort	Ascertainment of exposure	Demonstration that outcome of interest was not present at start of study	Comparability of cohort on the basis of the design or analysis	Assessment of outcome	Was follow-up long enough for outcomes to occur	Adequacy of follow up cohorts	
Hong et al. (2016) [14]	1	1	1	1	1	1	1	1	7
Syed et al. (2016) [17]	1	1	1	1	1	1	1	1	7
Lee et al. (2018) [15]	1	1	1	1	1	1	1	0	6
Bumgamer et al. (2019) [16]	1	1	1	1	1	1	1	1	7
Enriquez et al. (2019) [10]	1	1	1	1	1	1	1	1	7
Marano et al. (2020) [4]	1	1	1	1	1	1	1	1	7

Most of the patients involved in the included studies had bileaflet MVP. Premature ventricular contraction, NSVT, and VT with PM origin were common in most of the included studies. From one study, PVC-triggered VF occurred in 16% of patients [10]. During the electrophysiological study, PVC originating from PM or fascicular may serve as a trigger for VF in bileaflet MVP [17]. The acute success rate of CA varies between studies. The acute success rate for VA in the MVP group was 60% to 94%, depending on the type of VA. Three studies showed that the acute success rate of CA in the non-MVP group was higher than in the MVP group [10,14,15]. PM PVCs were less likely to have successful ablation procedures [10,16]. During the follow-up period, three studies reported the recurrence of VAs in MVP and non-MVP groups. Those studies showed that the MVP group had a higher percentage of VAs recurrence compared with the non-MVP group [10,14,15].

The basic demographics of the included studies and a summary of the included studies are presented in Tables 2, 3, respectively. A total of 1,257 patients were included in the analysis. The patients from included studies were generally female. The bileaflet was found more common compared with a single leaflet. PM origin of VAs was commonly reported from the included studies. The QRS duration, QTc duration, and left ventricular ejection fraction were measured in some studies. Two studies assessed the presence of MAD [4,16]. Most of the patients had mitral regurgitation with varying degrees between studies. Two studies reported subjects with severe MR [14,16]. Patients with MVP and VAs tend to have bileaflet prolapse and moderate MR [16]. However, MR was not associated with PVC burden [14]. The PVC burden before CA was quite different between studies.

**Table 2:** Basic demographics of the included studies LVEF, left ventricular ejection fraction; ms, milliseconds; MVP, mitral valve prolapse; NSVT, non-sustained ventricular tachycardia; PVC, premature ventricular complex; VAs, ventricular arrhythmias; VF, ventricular fibrillation; VT, ventricular tachycardia

First Author (year of publication)	Group	Number of patients	Age, years	Female gender (%)	QRS duration, ms	QTc interval, ms	LVEF, %	Mitral annular disjunction, patients (%)	Mitral regurgitation, patient %	PVC Burden, %
Hong et al. (2016) [14]	MVP	112	59.1 ± 15.2	64 (57%)	-	-	61.1 ± 6.7	-	63% trivial & mild, 33% moderate, 4% severe	9.4 ± 10.8
	Non-MVP	952	60.6 ± 16.8	490 (51%)	-	-	60.4 ± 7.3	-	85% trivial & mild, 12% moderate, 2% severe	8.4 ± 10.5
Syed et al. (2016) [17]	MVP with cardiac arrest	6	31.5 (24.2 – 58.7	6 (100%)	94.4 ± 9.3	461.5 ± 33.1	-	-	16.67% trivial, 50% mild, 33.33% moderate	60.4 (10.5 – 693.2) per hour
	MVP without cardiac arrest	8	36.3 (21 – 54.5)	7 (87.5%)	92.6 ± 11.8	459.4 ± 20.8	-	-	16.67% trivial, 50% mild, 33.33% moderate	606.4 (0.3 – 1424.3) per hour
Lee et al. (2018) [15]	PM VAs with MVP	9	45.56 ± 19.08	7 (77.78%)	-	-	50.44 ± 11.8	-	-	-
	PM VAs without MVP	14	57.64 ± 19.11	0 (0%)	-	-	45.15 ± 12.04	-	-	-
Bumgarner et al. (2019) [16]	MVP with PVC/NSVT	23	52.1 ± 13.4	16 (70%)	96 ± 15	441 ± 29	58 ± 5	-	8.7% trivial, 21.74% mild, 4.35% mild to moderate, 56.51% moderate, 8.7% severe	-
	MVP with sustained VT/VF	20	57.5 ± 11.9	7 (35%)	115 ± 27	452 ± 54	57 ± 4	-	5% without MR, 10% trivial, 10% mild, 30% moderate, 45% severe	-
Enriquez et al. (2019) [10]	MVP	25	54.7 ± 15.7	16 (64%)	-	-	50.5 ± 11.8	-	-	24.4 ± 13.1
	Non-MVP	73	59.9 ± 15.6	19 (26%)	-	-	41 ± 16.3	-	-	28.3 ± 12
Marano et al. (2020) [4]	MVP with VT/VF after index ablation	5	44 ± 16	3 (60%)	99 ± 11	462 ± 42	55 ± 6	3 (60%)	20% trace, 80% mild	17 ± 2
	MVP without VT/VF after index ablation	10	53 ± 13	7 (70%)	95 ± 14	435 ± 18	56 ± 8	7 (70%)	30% trace, 60% mild, 10% moderate	20 ± 12

 

 

**Table 3:** Summary of the included studies AV, aortic valve; CA, catheter ablation; ICD, implantable cardioverter-defibrillator; LV, left ventricle; LVOT, left ventricular outflow tract; MV, mitral valve; MVP, mitral valve prolapse; NSVT, non-sustained ventricular tachycardia; PM, papillary muscle; PVC, premature ventricular complex; RFCA, radiofrequency catheter ablation; RV, right ventricle; RVOT, right ventricular outflow tract; TV, tricuspid valve; VA, ventricular arrhythmia; VF, ventricular fibrillation; VT, ventricular tachycardia

First Author (year of publication)	Type of study	MVP group & control group	Leaflet involvement in MVP, %	Type & origin of ventricular arrhythmias	Type of Catheter Ablation	Acute Success Rate of MVP group & control group, %	Follow-up period	Outcome after catheter ablation, %
Hong et al. (2016) [14]	Retrospective	112 patients in MVP group (16 patients underwent CA) & 952 patients in control group (89 patients underwent CA)	62% bileaflet and 38% single leaflet	PVC and NSVT. PM, left anterior fascicle, inferoseptal LV fascicle, LV lateral wall, MV annulus, right septum, LVOT, RVOT	RFCA	94% in MVP group & 98% in control group	Mean follow up was 3.45 ± 2.5 years	1.8% in MVP group and 0.2% in control group had VF. Both patients in MVP group had implantable ICD and CA. 7% death in MVP group and 7% death in control group during follow up.
Syed et al. (2016) [17]	Retrospective	6 patients in MVP group with cardiac arrest & 8 patients in MVP group without cardiac arrest. Both of the group underwent ablation	Bileaflet	PVC, NSVT, & VT. PM, fascicle, MV annulus, outflow tract, purkinje	RFCA	86% in MVP group	Median follow up was 768 (39 – 2583) days	36% patients underwent repeat CA
Lee et al. (2018) [15]	Retrospective	23 patients in PM VAs and 129 patients in VAs of other site. PM VAs group were further divided into MVP (9 patients) and non-MVP (14 patients) group	88.89% bileaflet and 11.11% single leaflet	PVC, NSVT, & VT. PM, RVOT, LV summit, aortic sinuses of valsava, infundibulum	RFCA	72% in PM VAs group and 78% in VAs of other site group. 66.67% in MVP group & 85.67% in non-MVP group	Median follow up was 24 months for PM group and 7 months for Focal VAs of other site group	The VA recurrence was 25% nn MVP group and 15.3% nn non-MVP group Exertional sudden death occurred in 12.5% from MVP group
Bumgarner et al. (2019) [16]	Retrospective	43 patients (30 patients underwent CA) in MVP group. MVP group divided into PVC/NSVT group and sustained VT/VF group.	51% bileaflet and 49% single leaflet	PVC, NSVT, VT, & VF. RVOT, TV annulus, PM, LV apex, MV annulus, AV subvalvular, LV lateral wall, LV septal wall	RFCA	66.7% in MVP group	Mean follow up was 2.48 ± 3.38 years after ablation	7% with successful ablation had VA recurrence and were followed by ICD implantation
Enriquez et al. (2019) [10]	Retrospective	25 patients with MVP and 73 patients without MVP. All patients underwent CA.	72% bileaflet & 28% single leaflet	PVC, NSVT, & VF. Papillary muscle, MV annulus, RVOT, aortomitral continuity, left coronary cusp	RFCA	84% in MVP group and and 89.04% in non-MVP group	Mean follow-up was 31.5 ± 15.1 months	The VA recurrence was 28.6% in MVP group and 23.1%in control group 20% patients from MVP group had repeat ablation in 8.8 ± 8 months after the index ablation Sudden death occurred in 4% from MVP group
Marano et al. (2020) [4]	Retrospective	15 patients with MVP further divided into VT/VF after index ablation and no VT/VF after index ablation. All patients underwent CA.	53.33% bileaflet and 46.67% single leaflet	PCV, NSVT, & sustained VT. LV anterior/posterior PM, LV anterior/posterior fascicle, RVOT, LVOT, basal anterolateral LV, basal inferoseptal LV, superolateral MV annulus, RV his	RFCA	86.67% in MVP group	Median follow up was 3406 (1875 – 6551) days	33% patients develop significant VT/VF after index ablation 60% patients who develop significant VT/VF after index ablation underwent repeat ablation 67% patients without VT/VF after index ablation underwent repeat ablation for symptomatic PVCs/NSVT

**Figure 2:**
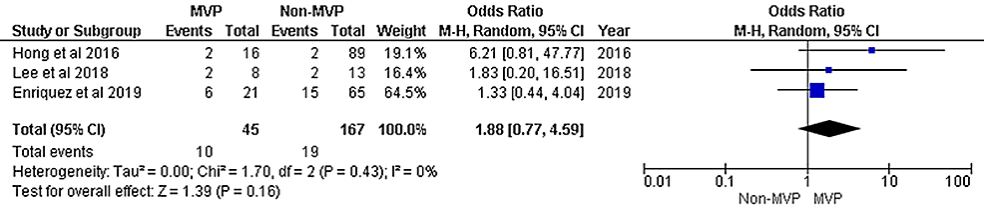
Forest plot of ventricular arrhythmia recurrence between groups MVP, mitral valve prolapse; CI, confidence interval; df, degree of freedom

The included studies for meta-analysis of VAs recurrence after CA are presented in Figure 2. A total of three studies were included. During the follow-up period of each study, 22.22% (10 patients) in the MVP group and 11.38% (19 patients) in the non-MVP group had VAs recurrence. Despite the higher recurrence in the MVP group, the recurrence rate between the MVP and non-MVP groups is not statistically significant (P-value = 0.16). No significant heterogeneity was observed from the included studies.

The medium-term and long-term outcomes were available from the included studies [4,10,14-17]. During the medium-term, some patients had repeated ablation for VAs. The rate of repeat ablation procedures varied from 12.5% to 36%. The reasons for repeat ablation procedure were cardiac arrest due to VF, persistent symptom, recurrent PVC, recurrent PMs PVC-induced VT/VF, and high-grade PMs PVC refractory to medical therapy [10,14,15,17]. Distinct origin of VAs was observed in patients requiring repeat ablation procedures [10,15]. However, one patient had sudden death at 17 months after index ablation [10] and another patient had SCD during exercise after repeated CA [15].

In the long term, the need for a repeat ablation procedure was quite high, reaching as high as 40%. The reasons for the repeat ablation procedure were symptomatic PVC/NSVT and sustained VT. All patients had different origins of VAs in the repeat ablation procedure. No SCD was reported [4].

Discussion

The issue regarding VAs associated MVP has been highlighted for many years. Once known as a benign condition, MVP has been studied for its association with VAs. Patients with younger age, previous syncope, more PVCs, PM fibrosis, and larger longitudinal MAD distance in the posterolateral wall assessed by cardiac magnetic resonance (CMR) have a higher risk of developing VAs [18]. CA emerges as one modality for the treatment of VAs associated with MVP. There was a higher VAs recurrence among patients with MVP after CA in the long-term follow-up period with varying degrees of acute success rate. Furthermore, a repeat ablation procedure was needed due to multiple reasons [4,10,14,15,17] and SCD can occur [15]. 

The VAs associated with MVP arise from the combination of the substrate and the trigger [3,6]. The substrate of VAs in LV may arise from endocardial fibrosis in the LV. The mechanical trigger of VAs may arise from mitral valve (MV) leaflet dumping in the diastolic period or traction of the PM [6,19]. Interestingly, a mutation in Filamin C may be involved as a proarrhythmic substrate in patients with bileaflet MVP [20,21]. A case report by Bains et al. reported a patient with Filamin C mutation underwent repeat CA after a previous successful CA [21]. The reason for repeat ablation was symptomatic PVCs or runs of NSVT and the VAs arose from different origins. Therefore, underlying gene mutation may result in recurrent VAs with different origins after successful CA.

From six included studies, all studies used RFCA and the VAs arising from PM were the most common origin. However, PM VAs had quite poor catheter stability and deep VA foci location compared with VAs from other sources [15,22]. On the other hand, CA using cryoablation emerged as an alternative to RFCA and may provide better stability [23]. But the real-world results remained variably [9]. In addition, chronic mechanical stress of MVP and mechanical stretch due to MAD results in PM hypertrophy and further protects deep VA foci from endocardial ablation [15]. Late VAs recurrence may result from arrhythmic foci not ablated during the initial CA.

On the other hand, there was a hypothesis of the progressive arrhythmic substrate as a reason for recurrent VAs in MVP [24]. Two included studies in the analysis also reported that the VAs observed during repeat CA were different from the VAs from the initial successful CA [4,10], supporting the progression of the local arrhythmic substrate rather than CA failure in VAs recurrence [24]. In addition, diffuse fibrosis rather than focal fibrosis was more common in older patients [6]. From the studies comparing MVP and non-MVP groups, the mean age of the MVP group was 59 ± 15.2, 45.56 ± 19.08, and 54.7 ± 15.7 years old [10,14,15]. Suggesting older age may also contribute to the development of recurrent VAs.

Another intriguing factor that might contribute to recurrent VAs after successful ablation was MAD. In terms of frequency of VAs, patients with MAD were reported to have a higher frequency of PVC and NSVT compared to patients without MAD [3]. The sensitivity and specificity of disjunction >8.5 mm to predict non-sustained VAs were 6.7% and 83%, respectively [8]. The presence of MAD was independently associated with the need for VAs ablation [24]. In addition, histologic findings also showed a significantly longer MAD in sudden death patients with MVP and LV fibrosis compared to patients without MVP [25]. Although there was some evidence regarding the association between MAD and MVP, there was no solid evidence of MAD as a significant factor in MVP patients with recurrent VAs after successful ablation. Two included studies reported MAD in their cohort. Both studies did not specify whether there was any recurrent VAs or not.

SCD could still occur following ablation. The proposed mechanism of SCD in MVP was the association between the substrate, trigger, and transient initiating event [26]. The fibrosis serves as a substrate for VAs and the MV leaflet dumping or traction of the PM serves as a trigger [3,6]. Physical and emotional stress acts as a transient initiating event. Catecholamine surge during stress and complex VAs may induce SCD [11,26]. From the included studies, sudden death was reported after CA [10,15]. One SCD occurred during exertion [15]. Progressive fibrosis, different PVC origin, and transient initiating event may be plausible mechanisms for SCD following successful ablation [3,6,26]. A study from Marano et al. reported that patients presenting with multifocal VAs origin or with sustained VT induced at the index electrophysiological study may develop hemodynamically significant VT/VF requiring repeat ablation and/or implantable cardioverter-defibrillator therapy [4].

From six included studies in the analysis, two studies reported MVP patients with recurrent VAs after surgical MV repair [4,10]. Although case reports showed surgical MV repair can suppress VAs in patients with MVP [27,28], a retrospective study showed the effect of MV surgery was not equal in reducing PVC burden in bileaflet MVP [29]. The PVC reduction was more prominent in younger patients. Moreover, the study only involved patients with bileaflet disease [29]. This finding suggests not only mechanical trauma but also arrhythmic substrate may play a role in patients with MVP [3]. Also, MV surgery followed by CA may not protect the patients from hemodynamically significant VAs [4].

There are several limitations to this study. All the included studies are retrospective studies and only half of the studies compare MVP and non-MVP. Further larger prospective studies with a big sample size are needed to evaluate CA as a therapy in VAs associated MVP. Small sample size may contribute to an insignificant result of the analysis. Since follow-up duration from most of the included studies are two years, a longer follow-up period is needed to evaluate the long-term benefit of CA and VAs recurrence. Only one study evaluates MAD and MAD may play an important part in the development of VAs. Further studies are needed to explore the mechanism of MAD in relation to VAs.

## Conclusions

Patients with MVP may develop VAs along the course of the disease and the most common origin of VAs associated with MVP is PM. CA, with varying degrees of acute success rate, emerges as a potential treatment for VAs associated with MVP. However, VAs in MVP patients with PM origin is more difficult to ablate successfully and the recurrence of VAs in MVP patients is inevitable in some patients. In addition, SCDs could still occur in MVP patients after CA. Older age during CA, genetic predisposition, deep arrhythmic foci, multifocal VAs origin, diffuse fibrosis, and MAD may contribute to the recurrence of VAs. Further studies, stratification, and evaluation are needed to prevent fatal outcomes in VA associated with MVP after CA.
